# Overexpression of the human DEK oncogene reprograms cellular metabolism and promotes glycolysis

**DOI:** 10.1371/journal.pone.0177952

**Published:** 2017-05-30

**Authors:** Marie C. Matrka, Miki Watanabe, Ranjithmenon Muraleedharan, Paul F. Lambert, Andrew N. Lane, Lindsey E. Romick-Rosendale, Susanne I. Wells

**Affiliations:** 1 Cancer and Blood Diseases Institute, Cincinnati Children's Hospital Medical Center and University of Cincinnati, Cincinnati, Ohio, United States of America; 2 NMR-Based Metabolomics Core Facility, Division of Pathology and Laboratory Medicine, Cincinnati Children's Hospital Medical Center, Cincinnati, Ohio, United States of America; 3 McArdle Laboratory for Cancer Research, University of Wisconsin School of Medicine and Public Health, Madison, Wisconsin, United States of America; 4 Center for Environmental Systems Biochemistry, Dept. Toxicology and Cancer Biology and Markey Cancer Center, Lexington, Kentucky, United States of America; University of Nebraska Medical Center, UNITED STATES

## Abstract

The DEK oncogene is overexpressed in many human malignancies including at early tumor stages. Our reported *in vitro* and *in vivo* models of squamous cell carcinoma have demonstrated that DEK contributes functionally to cellular and tumor survival and to proliferation. However, the underlying molecular mechanisms remain poorly understood. Based on recent RNA sequencing experiments, DEK expression was necessary for the transcription of several metabolic enzymes involved in anabolic pathways. This identified a possible mechanism whereby DEK may drive cellular metabolism to enable cell proliferation. Functional metabolic Seahorse analysis demonstrated increased baseline and maximum extracellular acidification rates, a readout of glycolysis, in DEK-overexpressing keratinocytes and squamous cell carcinoma cells. DEK overexpression also increased the maximum rate of oxygen consumption and therefore increased the potential for oxidative phosphorylation (OxPhos). To detect small metabolites that participate in glycolysis and the tricarboxylic acid cycle (TCA) that supplies substrate for OxPhos, we carried out NMR-based metabolomics studies. We found that high levels of DEK significantly reprogrammed cellular metabolism and altered the abundances of amino acids, TCA cycle intermediates and the glycolytic end products lactate, alanine and NAD^+^. Taken together, these data support a scenario whereby overexpression of the human DEK oncogene reprograms keratinocyte metabolism to fulfill energy and macromolecule demands required to enable and sustain cancer cell growth.

## Introduction

The human DEK proto-oncogene encodes a highly conserved chromatin-associated protein that is overexpressed in a wide range of human malignancies. DEK was originally identified in acute myeloid leukemia as a fusion protein with NUP214 [1], and was subsequently shown to be overexpressed at the mRNA and protein levels in various cancer types including squamous cell carcinoma (SCC) [2–7]. This oncoprotein modifies the structure of chromatin [8–12], and has corresponding nuclear functions in transcription [13–16], epigenetics [14, 15, 17], and mRNA splicing [18, 19]. Overexpression *in vitro* promoted cancer-associated phenotypes, such as cellular life span, proliferation, survival, and motility, depending upon cell types and experimental model systems utilized [6, 20–25]. Keratinocytes comprise 90% of the human epidermis and are the cells of origin for squamous cell carcinoma. We have previously shown that the overexpression of DEK stimulates proliferation and hyperplasia of NIKS, human keratinocytes, when engineered into 3D organotypic rafts that mimic stratified human epidermis [24]. Furthermore, such overexpression collaborated with the high-risk human papilloma virus (HPV) E6/E7 oncogenes and hRas to stimulate anchorage independent growth of keratinocytes *in vitro* and the development of squamous cell carcinoma (SCC) *in vivo* [22]. Finally, *Dek* knockout mice compared to wild type mice were protected from the growth of chemically induced skin papillomas [22], and head and neck (HN) SCCs in a HPV16 E7-driven transgenic murine tumor model [26]. Together, these data clearly demonstrate oncogenic DEK activities at early and late stages of carcinogenesis.

A major hurdle in neoplastic transformation is the ability of cells to meet the high bioenergetic and biosynthetic needs necessary to sustain cancer cell growth. It is well established that cancer cells shift to a pro-anabolic metabolism induced by oncogenes, such as *c-Myc* [27]. Most notable is the Warburg effect wherein cancer cells increase glycolysis and lactic acid fermentation when compared to their non-transformed counterparts [28]. An increase in glycolysis provides cancer cells with energy and heightened potential for biomass production from glycolytic intermediates [29]. Several glycolytic intermediates are important precursors for biomass production, including glucose-6-phosphate (G6P), fructose-6-phosphate (F6P), and glyceraldehyde 3-phosphate (GAP) via the pentose phosphate pathway (PPP). The PPP generates ribose for nucleotide biosynthesis, and NADPH via the oxidative branch of the PPP. NADPH is used to control oxidative stress via the glutathione peroxidase/glutathione reductase system [30]. F6P is involved in the synthesis of hexosamines. Dihyroxyacetone phosphate (DHAP) is the precursor of glycerol phosphate for glycerolipid synthesis, and glycerate 3-phosphate (3GP) is the precursor for serine and glycine production used in purine biosynthesis, as well as the production of pyruvate [31–33]. Cancer cells may also fuel their growth with glutamine that can be used as an amino acid for protein synthesis, as a carbon source for lipid synthesis and pyrimidine synthesis, and as a primary nitrogen donor for hexosamine and nucleotide synthesis. Glutamine supports anaplerosis by replenishing tricarboxylic acid (TCA) cycle intermediates used for macromolecule production in actively dividing cells. Metabolic adaptation to effectively support neoplastic proliferation and survival is a hallmark of cancer and involves the deregulation of multiple metabolic pathways through oncogene expression [33–35].

With regards to the human DEK oncogene, our recent transcriptome analyses of DEK-depleted HNSCC cells revealed a decrease in expression of numerous metabolic enzymes (S1A and S1B Fig) [36]. These enzymes were associated with multiple metabolic pathways including nucleotide synthesis, NAD^+^ metabolism, mTOR signaling, cholesterol synthesis, glycolysis, and glutathione production, all of which support cellular growth. This suggested DEK might regulate cellular metabolism, a function that has not been explored previously. Therefore, we set out to define DEK-regulated metabolism in HNSCC cells and non-transformed keratinocytes. To this end, DEK was first overexpressed in NIKS, an immortalized keratinocyte cell line and established model for normal human keratinocytes with regards to growth and differentiation characteristics and their ability to form stratified epidermis [24, 37]. Second, we utilized HNSCC cells (C-SCC1) that express intermediate levels of endogenous DEK thus allowing for additional DEK overexpression in malignant cells. We analyzed the resulting isogenic pairs of NIKS and C-SCC1 cells by Seahorse analysis to determine rates of glycolysis and oxidative phosphorylation. DEK overexpression was sufficient to increase baseline and maximum rates of lactic acid fermentation and maximum oxygen consumption in both cell types. In order to quantify small metabolites that accompany the observed metabolic alterations, we performed nuclear magnetic resonance (NMR)-based metabolomics on extracted metabolites from NIKS and C-SCC1 cells and their respective media. One-dimensional ^1^H (proton) NMR is a powerful tool that reliably and quantitatively detects small metabolites [38]. We found that DEK-overexpressing NIKS and HNSCC cells harbored an accumulation of glycolytic end products including lactate, NAD^+^ and alanine. An accumulation of TCA cycle intermediates was detected in HNSCC cells, but not in NIKS, suggesting differential utilization of TCA cycle intermediates for energy and macromolecule production. Taken together, we identify novel DEK functions in driving metabolic pathways that are associated with cancer-related energy and macromolecule production. Significantly, this occurs in keratinocytes and SCC cells, suggesting these effects are not dependent upon a cancer cell specific co-variant but are inherent to DEK overexpression.

## Results

### DEK overexpression increases cellular metabolism in the absence of proliferative gains

**Fig 1:**
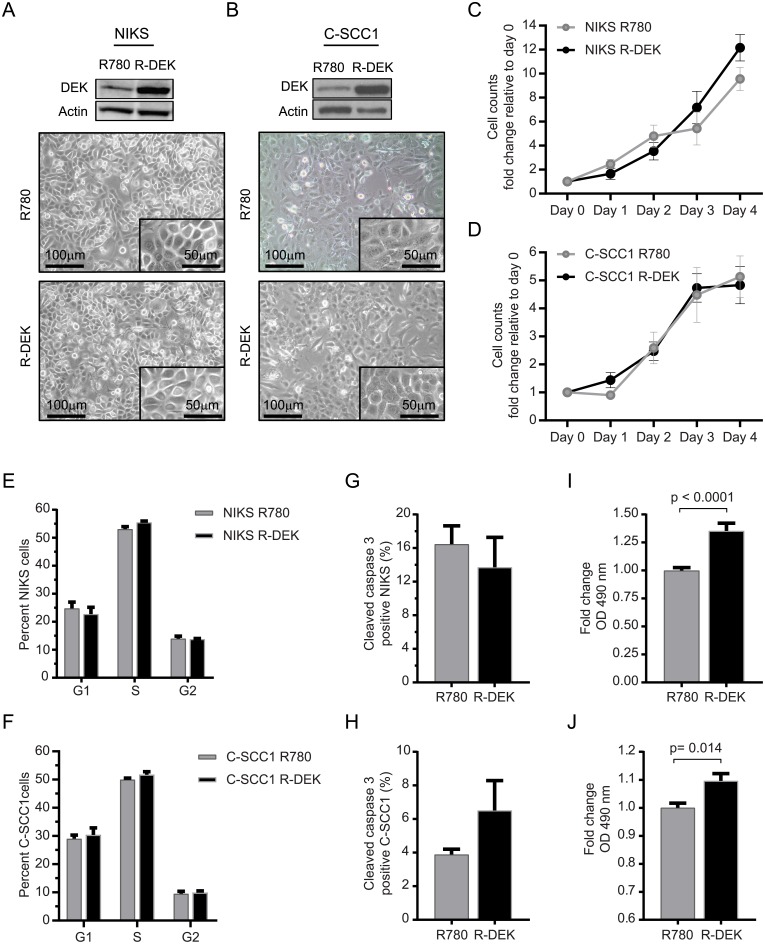
DEK overexpression increases cellular metabolic activity in NIKS and C-SCC1 cells in the absence of proliferative gains. (A-B) Western blot analysis validates DEK overexpression in NIKS (A) and C-SCC1 (B) cells with accompanying phase/light microscopy images taken at 10x magnification with a 20x inset of cells transduced with retroviral vector R780 control or R780-DEK (R-DEK). Images were taken on day 2 post plating. (C-D) Equal numbers of NIKS (C) and C-SCC1 (D) R780 or R-DEK cells were plated and counted over 4 days in 4 independent experiments. (E-F) Cell cycle profiles quantified by flow cytometry in NIKS (E) and C-SCC1 (F) R780 and R-DEK cells pulsed with EdU for 2 hours and stained with propidium iodide. The percentage of cells in each phase of the cell cycle was quantified from triplicate wells in three independent experiments. (G-H) Flow cytometry analysis of cleaved caspase 3 for the detection of apoptosis in NIKS (G) and C-SCC1 (H) R780 versus R-DEK cells. (I-J) Fold change in absorbance at 490 nm for MTS assay from 10,000 NIKS (I) or C-SCC1 (J) cells plated in a 96-well plate and measured 24 hours post plating at ~80% confluency. All error bars represent the standard error of the mean (SEM).

Our previous studies showed that DEK oncogene overexpression in NIKS [37] did not stimulate proliferation markers in monolayer cells, but increased proliferation and hyperplasia in 3D epidermis [24]. In order to determine whether DEK overexpression could promote metabolic activity, we transduced NIKS with either empty retroviral vector (R780) or with DEK-expressing vector R780-DEK (R-DEK) as previously described [22]. DEK overexpression was validated by western blot analysis relative to endogenous DEK levels, and did not affect overall cellular morphology (Fig 1A). Similar data were obtained upon DEK overexpression in a previously published HNSCC cell line CCHMC-HNSCC1 (C-SCC1) [26, 39] (Fig 1B). These cancer cells harbor moderate levels of DEK expression when compared to most other HNSCC cell lines, thus allowing for DEK upregulation beyond endogenous levels. As expected, DEK did not alter cellular growth as determined by cell counts over the course of 4 days (Fig 1C and 1D), cell cycle progression measured by EdU incorporation (Fig 1E and 1F), or apoptosis measured by caspase 3 cleavage (Fig 1G and 1H), in either NIKS or C-SCC1 cells. However, the tetrazolium-based MTS assay revealed increased oxidation of NADH to the key metabolic coenzyme NAD^+^, in R-DEK cells (Fig 1I and 1J). Thus, DEK overexpression stimulates metabolic activity in the absence of proliferative gains.

### DEK overexpression increases the rate of glycolysis and the maximum rate of oxidative phosphorylation

**Fig 2:**
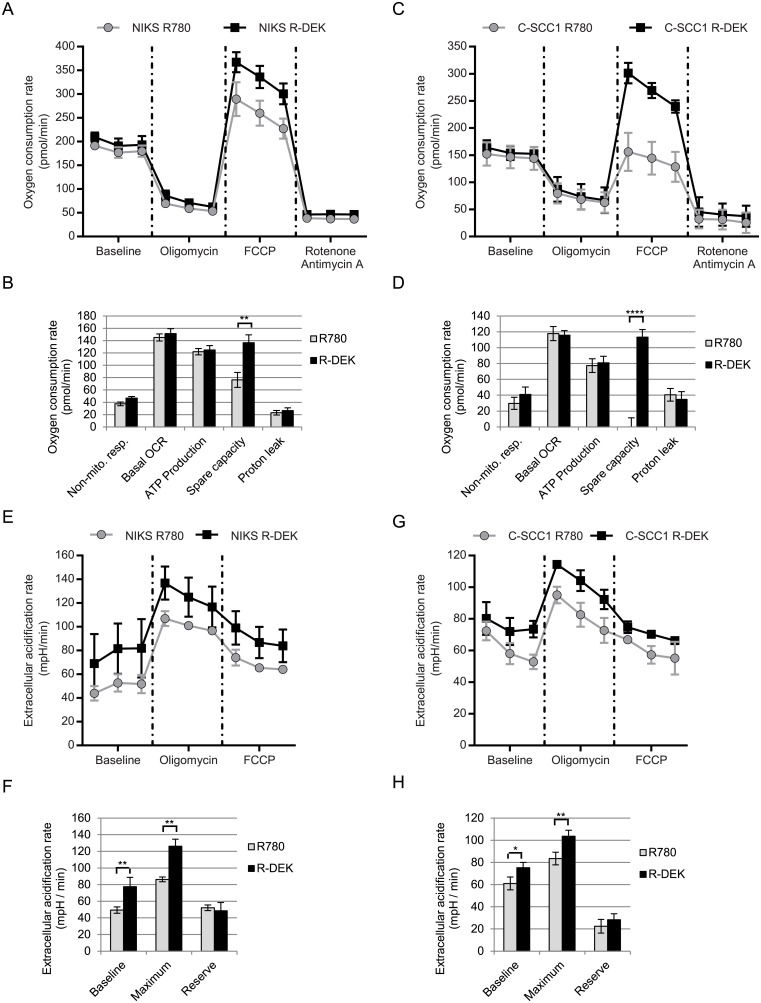
DEK overexpression increases glycolysis and the maximum rate of oxidative phosphorylation in NIKS and C-SCC1 cells. Seahorse XF24 Extracellular Flux Analyzer experiments using the mitochondrial stress test. (A-D) Quantification of oxygen consumption rate (OCR) measurements from 4 replicates of NIKS (A) and C-SCC1 (C) R780 and R-DEK samples taken three times at baseline and after treatment with the following pharmacological inhibitors of metabolism: oligomycin (ATP synthetase inhibitor), FCCP (and uncoupling agent), and rotenone and antimycin A (electron transport chain inhibitors). (B and D) Calculations from the mitochondrial stress test were as follows: non-mitochondrial respiration = oxygen consumed after treatment with electron transport chain inhibitors (rotenone and antimycin A). Basal OCR = baseline OCR minus non-mitochondrial respiration. ATP production = baseline OCR minus OCR after ATP synthetase inhibitor (oligomycin). Spare capacity = max OCR minus baseline OCR. Proton leak = OCR after oligomycin treatment minus OCR with electron transport chain inhibitors (rotenone and antimycin A). (E-H) The extracellular acidification rate (ECAR) was quantified for NIKS (E-F) and C-SCC1 (G-H) transduced with R780 or R-DEK. Quantification of glycolysis was calculated for baseline, maximum potential, and reserve potential in NIKS (F) and C-SCC1 (H). Reserve ECAR was calculated by subtracting baseline ECAR from maximum ECAR (oligomycin treated). Error bars represent the SEM of the 4 replicates. Statistical significance was determined using a t-test. Where indicated *P ≤ 0.05, **P ≤ 0.01, and ***P ≤ 0.001.

To define DEK effects on metabolism directly, we measured extracellular acidification rates (ECAR) and oxygen consumption rates (OCR), a readout for glycolysis and oxidative phosphorylation (OxPhos) respectively, using the Seahorse XF24 Extracellular Flux Analyzer (Seahorse Biosciences, Billerica, MA). NIKS and C-SCC1 R780 and R-DEK cells were assayed under baseline conditions and following injection of oligomycin, carbonyl cyanide 4-trifluoromethoxy-phenylhydrazone (FCCP), and rotenone/antimycin A. Oligomycin is an ATP synthase inhibitor that prevents OxPhos and drives glycolysis to the maximum rate. FCCP is an uncoupling reagent that induces maximum respiration rates and decreases glycolysis to baseline rates. Rotenone and antimycin A are electron transport chain inhibitors that halt OxPhos and have no additional effects on glycolysis. Interestingly, while OCR at baseline was similar between R780 and R-DEK NIKS (Fig 2A) and C-SCC1 cells (Fig 2B), the OCR after FCCP injection was increased with DEK overexpression (Fig 2A–2D). Therefore DEK increases the spare OxPhos capacity, i.e. the maximum rate of respiration possible, without affecting ATP production from OxPhos at baseline (Fig 2B and 2D). An increase in the maximum respiration rate provides extra OxPhos potential that is expected to support high energy demands when cellular growth is stimulated. With regards to ECAR, DEK overexpression caused a 60% baseline increase in NIKS (Fig 2E and 2F), and a 25% baseline increase in C-SCC1 cells (Fig 2G and 2H). DEK also increased the maximal achievable rate of glycolysis without increasing the glycolytic reserve for each cell type (Fig 2F and 2H). Overall, DEK increased baseline and maximum achievable glycolytic rates and increased the spare capacity for OxPhos in both cell types.

### Metabolic end products of aerobic glycolysis accumulate in DEK overexpressing NIKS

**Fig 3:**
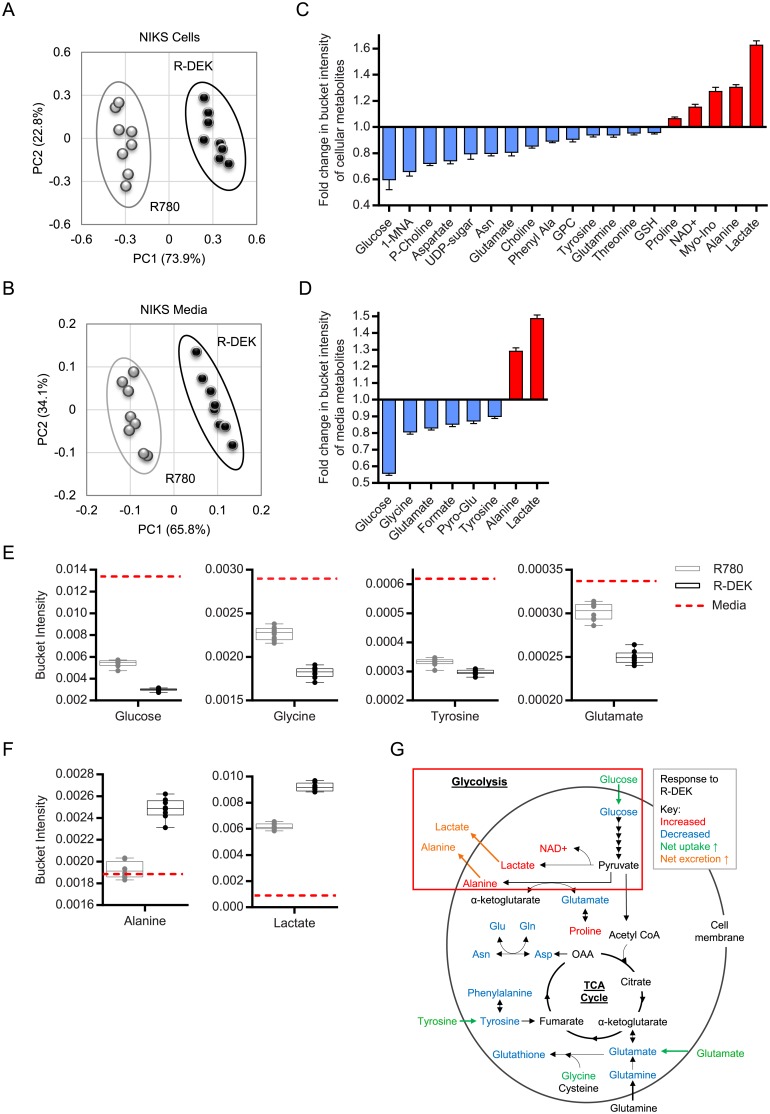
DEK overexpression increases the metabolic end products of glycolysis and the utilization of amino acids in keratinocytes. (A-B) Principal components analysis (PCA) scores plots of NIKS generated from normalized bucket intensities for 8 replicates showing separation based on metabolite presence between NIKS R780 (grey) and R-DEK (black) cells (A) and in their respective conditioned media (B). (C-D) Fold change in bucket intensities for each metabolite that was significantly different between R780 and R-DEK cells (C) and in their respective conditioned media (D) is arranged by magnitude of change. Metabolites in red are increased by DEK overexpression and metabolites in blue are decreased by DEK overexpression. Error bars represent the SEM in fold change of the 8 R780-DEK samples relative to the mean of the R780 controls. (E-F) The bucket intensity of metabolites averaged from triplicate samples of unconditioned media (dashed red line) were compared to R780 (grey) and R-DEK (black) conditioned media samples to identify metabolites that are decreased (E) and increased (F) compared to unconditioned control media. (G) Metabolic pathway schematic highlighting metabolites identified by NMR which were differentially regulated by DEK overexpression. The pathway analysis reveals many of metabolites increased upon DEK expression are products of aerobic glycolysis. Abbreviations: 1-MNA = 1 methylnicotinamide, p = phospho, Asn = asparagine, Phe = phenylalanine, GPC = glycerophosphocholine, NAD^+^ = nicotinamide adenine dinucleotide, Myo-ino = myo-inositol, Glu = glutamate, Gln = glutamine, α-keto = α-ketoglutarate.

The Seahorse data implicated DEK in the regulation of glycolysis and TCA cycle related metabolism. Therefore we utilized NMR-based metabolomics for in depth analyses of DEK-regulated metabolism. R780 and R-DEK NIKS and their respective media were collected on day 2 post plating, when cell numbers were indistinguishable (Fig 1C and 1D) and cells were at equal sub-confluency (Fig 1A and 1B, lower panel). Principal component analysis (PCA) separated DEK overexpressing cells (Fig 3A) and their respective media (Fig 3B) from the control cells and media. The significant differences spectra (SDS) identified regions of the NMR spectra that differed significantly between R-DEK versus R780 cells and media samples (S2A and S2B Fig). Thus, the data revealed that high DEK expression is sufficient to alter cellular metabolite profiles. DEK overexpression significantly altered 19 intracellular metabolites causing fold changes in bucket intensity ranging from 0.6–1.6 (Fig 3C and S2C Fig). Increased intracellular metabolites included NAD^+^, in line with the MTS assay (Fig 1I), along with proline, myo-inositol, alanine, and lactate. Decreased metabolites included glucose, 1-methylnicotinamide (1-MNA), phosphocholine (p-choline), aspartate, UDP-sugars, asparagine, glutamate, choline, phenylalanine, glycerophosphocholine (GPC), tyrosine, glutamine, threonine, and glutathione. We also detected significant changes in metabolite presence in the media of DEK overexpressing NIKS, including 6 metabolites that were significantly decreased and 2 metabolites that were significantly increased as determined by the SDS (Fig 3D and S2D Fig). The 6 metabolites decreased were glucose, glycine, glutamate, formate, pyroglutamate (a spontaneous breakdown product of glutamine), and tyrosine. Glucose, glutamate, and tyrosine were lower in both the cells and the media suggesting intracellular consumption and uptake from the media. Two metabolites that were higher in the media were lactate and alanine, which were also increased in the cells suggesting intracellular production and excretion.

In order to better quantify consumption, production, uptake and excretion for each metabolite, we compared bucket intensities present in the R780 and R-DEK media samples to the bucket intensities detected in F-media alone (unconditioned media). Net uptake into both R780 and R-DEK NIKS was observed for glucose, glycine, tyrosine, glutamate (Fig 3E) and pyroglutamate (S2E Fig) but with greater uptake into the R-DEK NIKS. Greater uptake along with an intracellular decrease in glucose, glutamate and tyrosine (Fig 3C) suggests higher rates of consumption for these metabolites by R-DEK NIKS. In reverse, lactate and alanine levels were increased in R-DEK cells and increased in the media versus R780 (Fig 3F), thus suggesting higher rates of production and excretion. The metabolites regulated by DEK overexpression are known participants in several metabolic pathways associated with cellular maintenance and growth. These include (1) glutaminolysis through glutamine and glutamate; (2) cell membrane maintenance through myo-inositol, GPC, choline, and p-choline; (3) cellular redox state through glutathione; (4) nucleotide synthesis through glycine, aspartate, glutamine, and formate; (5) protein synthesis through numerous amino acids; (6) one-carbon metabolism through glycine, and 1-MNA; and (7) glycolysis through glucose, lactate, alanine and NAD^+^; (illustrated in S2F Fig). However, by far the most striking difference in the R-DEK NIKS versus R780 was increased uptake and consumption of glucose, and the concomitant production of lactate, alanine, and NAD^+^. These metabolites include the substrate and end products of glycolysis implying that DEK promotes the conversion of glucose-derived pyruvate to lactate and alanine (Fig 3G). Therefore, the NMR metabolomics data validated increased ECAR measured by Seahorse analysis, and revealed a Warburg-like phenotype induced by DEK overexpression in immortalized keratinocytes.

### Metabolic end products of aerobic glycolysis accumulate in DEK overexpressing head and neck cancer cells

**Fig 4:**
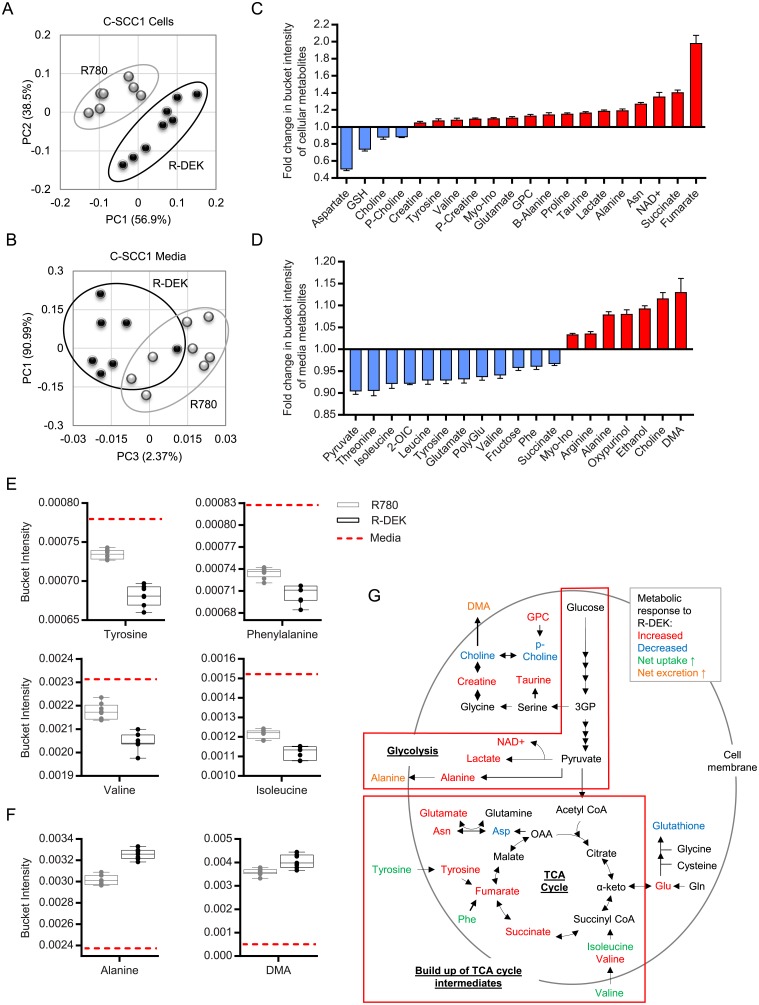
DEK overexpression in HNSCC cells increases glycolytic end products and TCA cycle intermediates. (A-B) PCA scores plot of C-SCC1 generated from normalized bucket intensities showing separation by metabolite presence between C-SCC1 R780 and R-DEK cells (A) and media (B). (C-D) Fold change in bucket intensities for each significantly changed metabolite between R780 and R-DEK cells (C) and media (D) arranged by magnitude of change. Error bars represent the SEM in fold change of the R780-DEK samples relative to the mean of the R780 controls. (E-F) The bucket intensity of metabolites in the unconditioned media (dashed red line) were compared to R780 (grey) and R-DEK (black) conditioned media samples to identify metabolites decreased (E) and those increased compared to control unconditioned media (F). (G) Metabolic pathway analysis highlighting metabolites identified by NMR that are differently regulated upon DEK overexpression. The metabolites identified are involved in various metabolic pathways including choline metabolism, protein and nucleotide synthesis, cellular redox state, aerobic glycolysis, and the TCA cycle. Abbreviations: p = phospho, Asn = asparagine, Phe = phenylalanine, GPC = glycerophosphocholine, NAD^+^ = nicotinamide adenine dinucleotide, Myo-ino = myo-inositol, Glu = glutamate, Gln = glutamine, α-keto = α-ketoglutarate, 2-OIC = 2-oxoisocaproate, Poly-Glu = polyglutamate, DMA = dimethylamine.

We next determined the effects of DEK overexpression on metabolic profiles in transformed C-SCC1 HNSCC cells. As was observed in the NIKS, high DEK expression was sufficient to alter metabolite presence both intracellularly and in the media based on PCA analysis (Fig 4A and 4B) and SDS plots (S3A and S3B Fig). Fold changes in intracellular metabolite bucket intensities spanned from 0.5 to 2.0 for 20 metabolites identified as significantly changed with DEK overexpression (Fig 4C and S3C Fig). Most of the 20 intracellular metabolites altered with DEK expression were increased and included the amino acids tyrosine, valine, glutamate, alanine, proline, and asparagine along with other non-amino acid metabolites creatine, p-creatine, taurine, myo-inositol, GPC, NAD^+^, fumarate and succinate, with the last two being TCA cycle intermediates. The only metabolites that decreased with DEK overexpression were aspartate, GSH, choline, and p-choline in the C-SCC1 cells. Fold changes in media metabolite bucket intensities were modest and spanned from 0.9 to 1.13 for 19 metabolites (Fig 4D and S3D Fig). Most metabolites in the media were decreased by DEK overexpression and included mainly amino acids, along with pyruvate, 2-oxoisocaproate, fructose, and succinate (Fig 4D). Seven metabolites in the media were increased by DEK overexpression and included myo-inositol, arginine, alanine, oxypurinol, ethanol, choline, and dimethylamine (DMA) (Fig 4D). Comparison in bucket intensities of significantly changed media metabolites to those in unconditioned media showed that DEK alters the uptake and excretion of various metabolites (Fig 4E and 4F and S3E Fig). For instance, bucket intensities of alanine and DMA were higher in the R-DEK and R780 samples compared to unconditioned media; albeit greater in the R-DEK samples suggesting DEK increases their production and excretion (Fig 4F). As expected from the Seahorse ECAR data, among DEK-induced metabolites in the HNSCC cells were once again lactate, alanine and NAD^+^ (Fig 4C) along with the excretion of alanine (Fig 4F), suggesting increased glycolysis. These data demonstrate that DEK overexpression can further upregulate aerobic glycolysis and lactate production in C-SCC1 cells that are already transformed (Fig 4G).

### DEK overexpression stimulates the accumulation of TCA cycle intermediates in HNSCC cells

The most prominent difference in R-DEK compared to empty vector control C-SCC1 cells was an increase in the TCA cycle intermediates fumarate and succinate (Fig 4C), and significant changes in amino acids that can feed into the TCA cycle such as tyrosine, phenylalanine, valine, isoleucine, aspartate and glutamate (Fig 4C and 4G). R-DEK cells had increased tyrosine and valine intracellularly (Fig 4C) as well as increased uptake from the media (Fig 4E). Isoleucine and phenylalanine were also taken up from the media more so in the R-DEK C-SCC1 cells (Fig 4E) although not altered in presence intracellularly. While the derivation and fate of these metabolites is unknown, they are all capable of being converting into, or having been converted from, TCA cycle intermediates (Fig 4G). Together these metabolite changes are likely reflections of alternative usage of TCA cycle intermediates for energy production and anabolic pathways such as lipid, protein and nucleotide synthesis. They may also be responsible for the large increase in spare capacity of OxPhos in the C-SCC1 cells. A map of DEK-regulated metabolites and the associated metabolic pathways are shown in S3F Fig.

### DEK overexpression affects similar and unique metabolic pathways in NIKS and C-SCC1 cells

**Fig 5:**
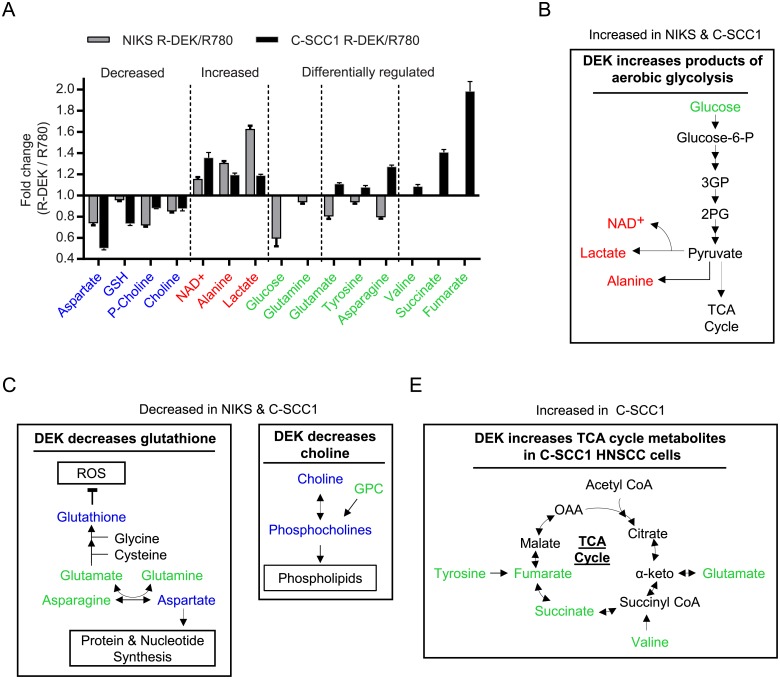
DEK overexpression drives glycolytic and glutathione pathways in NIKS and C-SCC1 cells, but uniquely stimulations TCA cycle intermediate accumulation in C-SCC1 cells. (A) Fold change in R-DEK compared to R780 bucket intensities for metabolites identified in NIKS (grey bars) and C-SCC1 cells (black bars). Metabolite names labelled in blue are decreased and those in red are increased in both cell lines. Metabolites labelled in green are either jointly but differentially regulated or only regulated in one cell line; in either case, the metabolite is higher in the C-SCC1 cells and/or lower in the NIKS with DEK overexpression. (B-E) Metabolites regulated in one or both normal/cancer cells are indicated within known associated metabolic pathways. (B) Metabolite products of aerobic glycolysis are increased in both cell lines (red) with an increase in glucose uptake in the R-DEK NIKS. (C) Glutathione and aspartate are decreased in both cell lines while metabolites surrounding this pathway are decreased in NIKS (green) and increased in C-SCC1 cells. (D) Choline and p-choline are decreased in both cell lines while GPC was differentially regulated. (E) Metabolites in and surrounding the TCA cycle are increased in the C-SCC1 cells (green) and either unchanged or decreased in the NIKS. Abbreviations: p = phospho, GPC = glycerophosphocholine, NAD+ = nicotinamide adenine dinucleotide, α-keto = α-ketoglutarate, OAA = oxaloacetate, ROS = reactive oxygen species.

In order to define metabolic pathways that are shared or unique between NIKS and C-SCC1 cells, we compared fold changes in metabolites induced by DEK overexpression between the cell lines (Fig 5A and S4A and S4B Fig). In NIKS and C-SCC1 cells, DEK increased the levels of intracellular lactate, alanine, and NAD^+^ (Fig 5A), metabolites closely related to increased glycolysis (Fig 5B). Lactate production was further validated in both cell lines by a colorimetric assay (S4C Fig). DEK expression also consistently decreased choline, p-choline, aspartate, and GSH (Fig 5A), metabolites involved in choline metabolism, protein and nucleotide synthesis, and oxidative stress reduction (Fig 5C). DEK overexpression differentially regulated glutamate, tyrosine, asparagine, glucose, glutamine, valine, succinate, and fumarate between the cell lines (Fig 5A). All were increased in C-SCC1 cells, and decreased in NIKS. While the cause for this differential abundance remains unclear, these metabolites are closely linked to the TCA cycle (Fig 5E) which is vital for energy and macromolecule production. Therefore, DEK uniquely regulated the presence of TCA cycle intermediates in the C-SCC1 cells, consistently decreased GSH, choline, p-choline, and aspartate, and consistently increased glycolytic end products. In conclusion, the presence of TCA cycle intermediates and substantially decreased GSH may be a reflection of the oncogenic effects of DEK specific to cancer cells. In contrast, elevated aerobic glycolysis may be a ubiquitous consequence of DEK overexpression that can promote and sustain uncontrolled cellular growth.

## Discussion

The Warburg effect is a metabolic hallmark of virtually all cancer cells, characterized by excessive conversion of glucose to lactate even in the presence of oxygen [40, 41]. In contrast to an earlier notion that the Warburg effect is accompanied by a decline in OxPhos, more recent studies have shown that mitochondrial function including OxPhos is in general not impaired and in fact required by cancer cells [42–44]. In HNSCC, there is plasticity of metabolic states in which the leading edge of the tumor, with more oxygen availability, relies preferentially on OxPhos while the inner compartment prefers glycolysis [45]. The shift and preference of metabolic pathways is often regulated by oncogene expression [46]. While most of the metabolic studies of oncogenes have been conducted in cells that were already transformed, the consequences of oncogene expression in normal or immortalized cells are less well understood, particularly in human keratinocytes. Insights into early metabolic responses to oncogene expression offer potential avenues for prophylactic SCC prevention, either through metabolic enzyme modulation or dietary means. Here we overexpressed the human DEK oncogene and compared metabolic consequences in immortalized versus transformed keratinocytes. Interestingly, in both NIKS and C-SCC1 cells, DEK increased glycolysis and lactic acid fermentation, and the maximum potential rate of oxidative phosphorylation. Importantly, this occurred in the absence of proliferative gains or cell death, as quantified by cell counts and flow cytometric analyses of cell cycle progression and apoptosis.

The NMR metabolomics study revealed that DEK overexpression can significantly de-regulate numerous metabolites in cells and media with fold changes ranging from +/- 1.1–2.0. In the C-SCC1 media samples, subtle changes were detected for several metabolites that nonetheless were statistically significant. We cannot fully rule out the possibility that small differences in cellular growth could be responsible for these metabolic responses. However, the growth of both NIKS and C-SCC1 cells overexpressing DEK was exceedingly similar to that of their respective control cells on day 2 post plating, when samples were collected for NMR. Therefore, these small changes are likely attributed to DEK overexpression and not proliferation. In subsequent analyses, we focused on those metabolites which displayed the highest fold changes and were similarly regulated in both cell lines thus suggesting broader biological impact. Importantly, the extent of regulation of glucose and lactate between R-DEK versus R780 samples was in line with previous reports where similar regulation was linked to physiologically relevant outcomes. For example, in pancreatic ductal adenocarcinomas that are strictly dependent on Kras mutations, mutated Kras caused a 1.2–1.5 fold increase in glucose consumption and a 1.3–1.4 fold increase in lactate production that was sufficient to increase hexosamine and nucleotide synthesis that contributed to anabolic metabolism [47]. We measured a 1.4 fold increase in glucose consumption in the NIKS (Fig 3C) and a 1.2–1.6 fold increase in lactate production in NIKS and C-SCC1 cells (Figs 3C and 4C) similar to the above Kras induced fold changes. The production of lactate has been shown to contribute to the tumor microenviroment and was sufficient to induce VEGF and Arg1 expression via HIF1alpha to promote angiogenesis and macrophage polarization [48–50]. Furthermore, elevated lactate concentrations in tumors were also associated with the subsequent development of nodal or distant metastases in head-and-neck cancer patients [51]. In addition to such biological consequences, the extent of metabolic deregulation caused by DEK overexpression is similar to reported changes in cancer versus normal tissue. For instance, an NMR study that detected metabolites in esophageal carcinoma compared to normal mucosa found a 1.15–1.35 fold increase in glucose consumption in tumor samples [52]. Another study detected a 1.2 fold increase in glucose consumption and a 1.3 fold increase in lactate production in the serum of esophageal adenocarcinoma patients when compared to healthy controls [53]. In this same study, fold changes of ~20 metabolites ranged from 1.1–1.6, with an average of 1.3. Thus, the reported extent of metabolite deregulation either induced by oncogene activation or in human tumors is similar to that induced by DEK overexpression, suggesting DEK overexpression can alter metabolism to support tumor growth.

The activation of cellular oncogenes such as *MYC* and *HIF1α* can de-regulate the transcriptional expression of metabolic enzymes like GLUT1/3, HK1/2 and lactate dehydrogenase A (LDHA) which are important drivers of glycolysis [54, 55]. Interestingly, DEK knockdown repressed LDHA expression according to a previously published RNA sequencing study (S1A and S1B Fig), and repressed other key metabolic enzymes that drive glucose metabolism in HNSCC such as FASN [56, 57], PDK1 [58], PKM2 [59–61], and HK2 [62]. It is possible that DEK overexpression transcriptionally activates specific metabolic enzymes and/or drivers of metabolism such as mTOR, which is known to activate several anabolic pathways including protein and lipid synthesis. Interestingly, mTOR was identified as a transcriptional target of DEK in the RNA sequencing study (S1 Fig), and DEK has been linked to mTOR activation in a published study. Therein, expression of the DEK-NUP214 fusion protein identified in acute myeloid leukemia increased mTORC1 activity in myeloid cells, leading to increased protein synthesis and proliferation that was attenuated by an mTOR inhibitor [63]. In contrast to DEK overexpression in our study, DEK-NUP214 expression decreased lactate production without affecting glucose uptake, suggesting a shift from glycolysis to oxidative phosphorylation. While it is possible that DEK overexpression, like DEK-NUP214 expression, promotes mTOR signaling to deregulate metabolism, these results indicate that DEK overexpression versus expression of the DEK-NUP214 fusion protein elicit distinct metabolic consequences in hematopoietic cells versus keratinocytes.

In further support of transcriptional regulation of metabolic enzymes by DEK, we identified a strong increase in DMA in the medium of DEK overexpressing C-SCC1 cells (Fig 4D). DMA is produced by the enzyme dimethylarginine dimethylaminohydrolase (DDAH1), and RNA sequencing studies demonstrate strong repression of DDAH1 gene expression in two HNSCC1 cell lines in response to DEK depletion (S1 Fig). These data suggest that DEK promotes the transcription of multiple metabolic enzymes, and in this study we observe aberrant regulation of metabolites known to be controlled by enzymes transcriptionally regulated by DEK.

In NIKS and C-SCC1 cells, NMR-based metabolomics identified shared metabolic signatures related to DEK overexpression. These included an increase in metabolites derived from glucose including lactate, alanine, NAD^+^ and myo-inositol (Figs 3C and 4C). With regards to myo-inositol, a previous NMR study revealed significantly increased myo-inositol in HNSCCs from three different anatomical locations relative to normal human oral keratinocytes [64]. Furthermore, in HNSCC, the H^+^-myo-inositol transporter SLC2A13 was consistently increased in sphere-forming cells derived from primary oral SCC specimens suggesting myo-inositol transport is linked to oral SCC stem cells [65]. We found DEK overexpression is sufficient to increase myo-inositol, a metabolite that is upregulated in HNSCC and its stem cells. This suggests DEK may regulate cancer stem cell metabolism which is in line with data from a breast cancer study showing DEK is required to sustain the growth of a stem cell population [23].

Additional shared metabolites identified by NMR between cell lines are lactate, NAD^+^ and alanine (Figs 3C and 3D, 4C and 4D). The NMR data in conjunction with functional metabolism data based on Seahorse analysis (Fig 2E–2H), and lactate assays (S4C Fig) have established DEK promotes glycolysis and lactic acid fermentation. In NIKS, glucose was the most decreased metabolite in cells and in the media of R-DEK relative to empty vector samples, strongly suggesting DEK driven glucose metabolism and glycolysis are responsible for the observed increases in lactate and alanine. However, we cannot rule out the possibility that glutamine and other amino acids sources contribute to the production of lactate and alanine. In addition to driving baseline glycolysis, DEK also increased the maximum glycolytic potential in both NIKS and C-SCC1 cells. An increase in maximum glycolysis suggests DEK overexpressing cells are more fit to handle high proliferative rates. Increased glycolysis in HNSCC cells has been well documented in the literature [64, 66–69]. Here, we note that glycolysis in C-SCC1 cells can be even further stimulated by DEK overexpression (Fig 2G and 2H). Corresponding decreases in OxPhos were not observed. In fact, DEK overexpression increased the maximum achievable OCR in NIKS (Fig 2A), and in C-SCC1 cells (Fig 2C), thus increasing their potential to produce energy from both lactic fermentation, and OxPhos if required.

DEK overexpression promoted the consumption of metabolites for nucleotide, protein and/or GSH synthesis. Glutamine, glutamate, aspartate, formate and glycine contribute to nucleotide synthesis by donating carbon and/or nitrogen atoms to purine and pyrimidine rings in de novo synthesis. In NIKS, DEK decreased intracellular glutamate, glutamine, and aspartate while promoting glycine uptake and repressing formate export (S2E Fig). Glycine can also be utilized in one carbon metabolism via methylenetetrahydrofolate reductase (MTHF) and the methylation of proteins, nucleotides and lipids (S2F Fig). Glutamate and glycine along with cysteine, produces GSH, a key cellular redox regulator, which was decreased with DEK overexpression in NIKS (Fig 3C) and C-SCC1 cells (Fig 4C). The observed decrease may reflect oxidation of GSH by reactive oxygen species, a possible indication of increased ROS in DEK overexpressing cells resulting from increased cellular metabolism. However, oxidized glutathione (GSSG) is not detected by NMR using our cellular extraction protocol, and therefore, GSSG production remains unproven. Interestingly, the decrease in GSH was far greater in the C-SCC1 cells, potentially caused by increases in fumarate in those same cells (Fig 4C). Fumarate can bind GSH and convert it to succinated glutathione, which depletes NADPH levels and increases ROS [70].

Another metabolic consequence of DEK overexpression was decreased intracellular presence of cell membrane components choline and p-choline, along with differentially regulated levels of GCP (Fig 5A and 5C). The decrease in choline compounds caused by DEK could indicate their increased incorporation into membranes or could be due to increased choline breakdown. While choline compounds are generally increased in other cancer types, and are considered biomarkers for breast cancer, we have observed a decrease in choline levels in C-SCC1 cells in line with another NMR study of oral SCC [71]. Therefore, DEK overexpression is sufficient to cause a decrease in choline levels in keratinocytes and SCC cells.

In conclusion, we have identified a new role for DEK in regulating cellular metabolism by increasing the rate of glycolysis and OxPhos potential, with a concomitant regulation of small metabolites involved in multiple anabolic pathways. This suggests that high DEK expression, observed in numerous malignancies including at early stages, may provide the required gains in energy and macromolecule production to enable uncontrolled cancer growth and progression. Future studies using stable isotope resolved metabolomics will define DEK-driven precursor-product relationships at the atomic level, in order to uncover key enzymes which may themselves be targeted for cancer prevention and treatment [72].

## Materials and methods

### Viral constructs and transductions

Human DEK was overexpressed using a retroviral R780 vector [22]. Cells were incubated with virus for 4 hours in medium containing 2 μg/mL of polybrene (Sigma Aldrich, product-9268), then washed and overlaid with fresh medium. Transduced cells were sorted for GFP expression on a BD FACS Canto analyzer and expanded as a polyclonal population.

### Cell culture

The spontaneously immortalized near-diploid human keratinocyte cell line (NIKS) [37] was maintained in F-media, which is 3 parts Dulbecco's modified Eagle's medium to 1 part Ham's F12 media (lifetechnologies, product-11765070) supplemented with the following components: 5% fetal bovine serum, 24 ug/mL adenine, 8.4 ng/mL cholera toxin (Millipore, product-227036), 10 ng/mL epidermal growth factor (Sigma Aldrich, product- e4127), 2.4 μg/mL hydrocortisone (Sigma Aldrich, product-h0888), 5 μg/mL insulin, 1% penicillin-streptomycin (Lifetechnologies), and 0.2% fungizone (Omegascientific). CCHMC-HNSCC1 (C-SCC1) head and neck cancer cells were cultured from an HPV positive, stage IV, tonsillar tumor obtained with IRB approval at the time of surgical resection [26]. These cells were used between passages 10–15. Both cell lines were plated on irradiated 3T3-J2 mouse fibroblasts and maintained in F-media. NIKS and C-SCC1 cells were cultured until 80% confluency when few (<5%) feeders remained on the plate.

### Western blot analysis

Cells were washed with PBS, and whole cell lysates were harvested with RIPA buffer (1% Triton, 1% deoxycholate, 0.1% SDS, 0.16 M NaCl, 10 mmol/L Tris pH 7.4, and 5 mmol/L EDTA) supplemented with a protease inhibitor cocktail (BD PharMingen- 554779) and analyzed as described previously [39]. The DEK primary antibody is from BD Biosciences used at a 1:1000 dilution. (BD Biosciences, product 610948), and pan-actin (1:20,000) is a gift from James Lessard. (Seven Hill Bioreagents, Cincinnati, OH, USA). Membranes were exposed to enhanced chemiluminescence reagents (Perkin Elmer).

### Cell counts

200,000 NIKS were plated into 6-well plates. Cells were counted every 24 hours using trypan blue and a BioRad cell counter TC-20. C-SCC1 cell counts were determined similarly except 50,000 cells were plated into a 12 well plate. Error bars represent the standard error of the mean (SEM) from triplicate experiments.

### Flow cytometry for cell cycle and apoptosis analysis

For cell cycle analysis, NIKS were grown to 70–80% confluency in 6 well plates and pulsed with 10 mM EdU for 2 hours before collection by trypsinization. Cells were prepared using the Click-iT^®^ EdU Alexa Fluor^®^ 647 Imaging Kit (Lifetechnologies, product-C10424) according to manufacturer specifications. DNA content was determined using propidium iodide. Apoptosis was determined using FITC active caspase 3 antibody kit (BD Biosciences, product 550480) using manufacturer instructions. Cells were analyzed on a BD FACS Canto analyzer in biological triplicates (BD Biosciences, San Jose, CA). A t-test was used to determine significance between R780 and R-DEK samples from the independent experiments.

### MTS assays

R780 empty vector or R-DEK cells were plated in 10 wells of a 96-well plate at a density of 10,000 cells per well. The cells were allowed to grow for 24 hours. PMS was added to the MTS (Promega product G1112) reagent at a 1:50 dilution. Two hours later the plate was read on a spectrometer at an absorbance of 490 nm. Blank media readings were subtracted from the reading. The data are from three independent experiments and the readings were averaged across all three. Significance was determined by t-test. Error bars represent the SEM from the three independent experiments.

### NMR-based metabolomics

#### Media collection and cell collection/extraction

One million cells were plated in a 10 cm plate, media was replaced after 24 and cells were collected 24 hours later. For C-SCC1 cells only, 5 mL of new media was added to cells 4 hours prior to collection. A total of 8 replicates of NIKS and C-SCC1 R780 empty vector control cells and R-DEK overexpression vector cells were collected at 80% confluency. Due to sample loss during the NMR process, C-SCC1 R780 cells and C-SCC1 R-DEK media had 7 replicates. 10 mL of media were collected before cell extraction and centrifuged at 3500 g for 20 minutes at 4°C. Three milliliters per sample were collected into two Eppendorf tubes for further processing. Intracellular metabolites were extracted using the methanol-water direct scraping technique as previously described with few modifications [73]. Briefly, after removing the growth media, cells were rinsed twice with 5 mL cold PBS on ice, then scraped with 1 mL of ice-cold 2:1 (v/v) methanol: water solution twice and transferred to a 2mL Eppendorf tube. After vortexing, the tubes were incubated on ice for 5 min and centrifuged for 5 min at 6000 *xg* at 4°C. The supernatant, *i*.*e*. polar extract, was transferred into a pre-weighed new 1.5mL Eppendorf tube. The samples were dried in a SpeedVac centrifuge for 2–4 hrs and stored at -20°C. Prior to data collection, the dried polar cell extracts were resuspended in 600 μL of NMR buffer (100 mM potassium phosphate (pH 7.3), 0.1% sodium azide, 1 mM trimethylsilylproprionate (TSP) in 100% D_2_O).

#### Media sample processing

On the day of the data collection, samples were thawed on ice and centrifuged 4000 *xg* for 5 min at 4°C. The 500 μL supernatant of media samples were placed onto pre-washed 3 kDa spin filters (NANOSEP 3K, Pall Life Sciences), and centrifuged at 10000 *x g* for 90 min at 4°C. The 400 μL of plasma filtrate was mixed with 200 μL of NMR buffer. In order to monitor the original metabolites supplemented to the cell, unconditioned media samples (F-media) were also prepared in the same manner. For NIKS, three replicates of F-media were prepared and for the C-SCC1s one sample was prepared since the replicates for NIKS showed no variation.

#### NMR spectroscopy acquisition and processing

All experiments were conducted using 550 μL samples placed in 103.5 mm x 5 mm NMR tubes (Bruker). One-dimensional ^1^H NMR spectra were acquired on a Bruker Avance II 600 MHz spectrometer. All data were collected at a calibrated temperature of 298 K using a three-pulse sequence based on the noesypr1d (or noesygppr1d) pulse sequence in the Bruker pulse sequence library. This pulse sequence provided water-suppression with good baseline characteristics. Experiments were run with 8 dummy scans (DS) and 256 (cell extract) and 128 (media) acquisition scans (NS) with an acquisition time (AQ) of 3.4 s and a relaxation delay (D1) of 2.0 s for a total repetition cycle (AQ+D1) of 5.4 s. The mixing time was 10 ms. The spectral width was 16 ppm, and 64K real data points were collected. Under automation control, each sample analysis took about 10 minutes for setup and 30 minutes for acquisition. During the 10 minute setup time, the temperature was monitored for equilibration. All FIDs were subjected to an exponential line-broadening of 0.3 Hz. Upon Fourier transformation, each spectrum was manually phased, baseline corrected, and referenced to the internal standard TSP at 0.0 ppm for polar samples using Topspin 3.5 software (Bruker Analytik, Rheinstetten, Germany). For two dimensional ^1^H-^1^H total correlation spectroscopy (TOCSY) data, a relaxation delay equal to 2 s, an isotropic mixing time of 80 ms at a B_1_ field strength of 10 kHz were used for 2048 data points with 128 scans per increment were acquired with spectral widths of 14 ppm.

### Metabolomics data analysis

#### Multivariate statistical analysis

Principal components analysis (PCA) was performed to look for metabolic differences using AMIX 3.9.15 software (Bruker Analytik, Rheinstetten, Germany). ^1^H NMR spectra were processed and analyzed with AMIX for PCA analysis. The spectra from 0.5 to 10.0 ppm, excluding the region of the residual water resonance (4.6 to 5.0 ppm) and methanol (3.36–3.38 ppm), were reduced by uniform binning to 995 buckets 0.01 ppm wide. The spectra were normalized to constant total spectral area. Prior to PCA analysis, the binned spectra were mean-centered with no scaling. PCA scores were exported and scores plots were generated using Microsoft Excel.

#### Univariate analysis

The spectral bucket intensities tables were further analyzed using a univariate approach, based on bin-by-bin differences between two groups. In order to identify the NMR spectral regions that are significantly different between the groups, ^1^H significant difference spectra (SDS) were generated [74, 75]. Pairwise differences within each bin were compared using Student’s t-test. The false discovery rate (FDR) was controlled at 0.05 level using the Benjamini-Hochberg method [76]. The SDS plot was generated by taking the mean difference of the buckets with significant differences between the two groups. Metabolites were assigned to those significant buckets based on the chemical shifts as described below. Fold changes were calculated by dividing the mean bucket intensity of one group over the mean of another: fold change = mean (R780-DEK)/ mean (R780). Error bars represent the SEM in fold change of the R780-DEK samples compared to the mean of the R780 controls. These were calculated by dividing each R780-DEK sample metabolite bucket intensity by the average R780 bucket intensity for the respective metabolite. There were 8 replicates per sample except for C-SCC1 R-DEK media and C-SCC1 R780 cells for which there were 7 replicates due to sample loss.

#### Spectral analysis/metabolites identification

Metabolites found in cell extract and media were assigned based on 1D ^1^H and 2D ^1^H-^1^H TOCSY NMR experiments. Peaks were assigned by comparing the chemical shifts and spin-spin couplings with reference spectra found in databases, such as the Human Metabolome Database (HMDB) [77], the Madison metabolomics consortium database (MMCD) [78], the biological magnetic resonance data bank (BMRB) [79], and Chenomx^®^ NMR Suite profiling software (Chenomx Inc. version 8.1)

### Lactic acid assay

A lactic acid determination kit was purchased from biovision (K607-100) to detect L-lactate in the media of NIKS and C-SCC1 cells. The manufacturer’s protocol was followed with the following specifications. 250K Cells were plated in a six well dish in triplicate wells overnight. The next day cells were overlaid with 1mL of 1x DMEM with no FBS and media samples were collected after 30 minutes. 20 μl of the media collected was loaded in duplicate in a 96 well plate and measured on a spectrometer at absorbance 570. Significance was determined using a student’s t-test to compare R780 to R-DEK samples from three independent experiments.

### Seahorse XFe96 metabolic flux analysis

Extracellular acidification rates (ECAR) and oxygen consumption rates (OCR) for NIKS and C-SCC1 R780 and R-DEK cells were determined using the Seahorse Extracellular Flux (XFe96) analyzer (Seahorse Bioscience, MA, USA). NIKS were seeded at 30,000 cells per well and C-SCC1 at 50,000 cells per well into XFe96 well cell culture plates and incubated for 16 h in F media at 37°C in a 5% CO_2_ humidified atmosphere. For ECAR, cells were washed in XF assay media. For OCR, cells were washed in XF assay media supplemented with pre-warmed 10 mM glucose, 1 mM Pyruvate, 2 mM L-glutamine adjusted to 7.4 pH. Cells were then kept in 175 μL/well of XF assay media at 37°C, in a non-CO2 incubator for 1 hr. During the cell incubation time, 9 μM oligomycin, 10 μM oligomycin, 9 μM FCCP, 10 μM rotenone, 10 μM antimycin A in XF assay media were loaded into the injection ports in the XFe96 sensor cartridge. Data sets were analyzed by XFe96 software and GraphPad Prism software, using FDR correction and t-test calculations. The experiment was performed 4 times with 4–6 replicates in each experiment. One experiment was chosen that best represented the trends seen across all experiments.

## Supporting information

S1 Fig DEK knockdown in HNSCC cell lines decreases the transcription of metabolic enzymes. (A) The fold change in relative transcript expression of genes encoding metabolic enzymes identified from a previously published RNA sequencing experiment in two HNSCC cell lines (UMSCC1 and UMSCC47) transduced with DEKsh or NTsh control lentiviral vectors [36]. The indicated genes were decreased with DEK knockdown in both cell lines and are involved in multiple metabolic pathways. (B) The fold change in mRNA encoding metabolic enzymes from DEKsh compared to NTsh cells represents the average between the two HNSCC cell lines.
(EPS)

S2 Fig DEK overexpression in NIKS significantly alters metabolite uptake and utilization. (A) Significant differences spectra of metabolites identified in NIKS cells and (B) NIKS media created from normalized bucket intensitieswith statistically significant differences. Significance is determined by Welch’s test with false discovery rate (FDR) correction. (C) Altered metabolites with corresponding spectral position (bin centers) and their fold changes as well as *p*-value (t-test) is reported for NIKS and (D) NIKS media. (E) Bucket intensities of metabolites in R780 and R-DEK samples after subtraction of the bucket intensities of metabolites in the unconditioned media. Metabolites in orange are increased, and metabolites in green are decreased in the R-DEK samples compared to unconditioned media. (F) A metabolic pathway schematic of significantly altered metabolites in NIKS with DEK overexpression. Metabolites intracellularly decreased (blue), increased (red), and those taken up from the media (green) or excreted into the media (orange) are identified within their associated metabolic pathways. DEK overexpression regulates metabolites involved in glutaminolysis, cell signaling and membrane maintenance, cellular redox state, nucleotide synthesis, protein synthesis, methylation, and glycolysis.
(EPS)

S3 Fig DEK overexpression in C-SCC1 significantly alters metabolite uptake and utilization. (A) Significant differences spectra of metabolites identified in C-SCC1 cells and (B) media created from normalized bucket intensities with statistically significant differences. Significance is determined by Welch’s test with FDR correction. (C) Altered metabolites with corresponding spectral position (bin centers) and their fold changes as well as *p*-value (t-test) is reported for C-SCC1 cells and (D) media. (E) Bucket intensities of metabolites in R780 and R-DEK samples after subtraction of the bucket intensities for each metabolite in the unconditioned media. Metabolites in orange are increased, and metabolites in green are decreased in the R-DEK samples compared to unconditioned media. (F) A metabolic pathway schematic of significantly altered metabolites in C-SCC1 cells with DEK overexpression. Metabolites intracellularly decreased (blue), increased (red), and those taken up from the media (green) or excreted into the media (orange) are identified within metabolic pathways.
(EPS)

S4 Fig DEK overexpression affects unique metabolites in NIKS and C-SCC1 cells but consistently increases lactate. (A) Fold change in bucket intensities for metabolites only identified in NIKS and (B) C-SCC1 cells. (C) Colorimetric lactic acid assay comparing lactate production from NIKS and C-SCC1 R780 and R-DEK cells.
(EPS)

## Abbreviations

1-MNA: 1-methylnicotinamide
2-OIC: oxoisocaproate
Ala: alanine
αKG: alpha ketoglutarate
Asn: asparagine
BCAA: branched chain amino acids
Cho: choline
DMA: dimethylamine
Gln: glutamine
Glu: glutamate
GPC: glycerophosphocholine
GSH: reduced glutathione
HNSCC: Head & Neck Squamous Cell Carcinoma
Myo-Ino: myo-inositol
NAD^+^: nicotinamide adenine dinucleotide
OAA: oxaloacetate
PCA: Principal Components Analysis
P-choline: phosphocholine
Phe: phenylalanine
Poly-Glu: poly glutamate
Pyr: pyruvate
Pyro-Glu: pyroglutamate
Suc: succinate
UDP: uridine diphosphate
